# Incidence and prognostic impact of *U2AF1* mutations and other gene alterations in myelodysplastic neoplasms with isolated 20q deletion

**DOI:** 10.1002/cam4.6300

**Published:** 2023-07-05

**Authors:** Martín I. Castillo, Villamón E. Ribate, Calabuig M. Muñoz, Sanz G. Santillana, Such E. Taboada, Mora E. Casterá, Calasanz M. J. Abinzano, Irigoyen A. Barranco, Collado R. Nieto, Vara M. Pampliega, M. L. Blanco, Álvarez S. de Andrés, Pérez J. de Oteyza, Bernal T. del Castillo, Granada I. Font, Jerez A. Cayuela, M. Díez‐Campelo, Abellán R. Sánchez, Solano C. Vercet, Tormo M. Díaz, Grupo Español de Síndromes Mielodisplásicos (GESMD)

**Affiliations:** ^1^ Servicio de Hematología Hospital Clínico Universitario de Valencia, Instituto de Investigación Sanitaria INCLIVA Valencia Spain; ^2^ Servicio de Hematología Hospital Universitario y Politécnico La Fe, Instituto de Investigación Sanitaria La Fe, CIBERONC – ISCIII Valencia Spain; ^3^ CIMA LAB Diagnostics Universidad de Navarra, CIBERONC – ISCIII Pamplona Spain; ^4^ Servicio de Hematología Consorcio Hospital General Universitario de Valencia Valencia Spain; ^5^ Servicio de Hematología y Hemoterapia del Hospital Universitario de Cruces Barakaldo Spain; ^6^ Servicio de Hematología Hospital de la Santa Creu i Sant Pau Valencia Spain; ^7^ NIMGenetics, Genómica y Medicina Madrid Spain; ^8^ Servicio de Hematología Hospital Universitario HM Madrid Madrid Spain; ^9^ Servicio de Hematología Hospital Universidad de Asturias, IISPA, IUOPA Valencia Spain; ^10^ Servicio de Hematología, Hospital Germans Trias i Pujol, Institut Català d'Oncologia, Institut de Recerca contra la Leucèmia Josep Carreras Universidad Autónoma de Barcelona, CIBERONC – ISCIII Bellaterra Spain; ^11^ Servicio de Hematología Hospital Universitario Morales Meseguer Murcia Spain; ^12^ Servicio de Hematología Hospital Universitario de Salamanca, CIBERONC – ISCIII Salamanca Spain; ^13^ Departamento de Bioquímica y Patología Molecular Hospital Clínico Universitario de Valencia, Instituto de Investigación Sanitaria INCLIVA Valencia Spain

**Keywords:** 20q deletion, myelodysplastic neoplasms, prognosis, quantitative allele‐specific PCR, *U2AF1* mutations

## Abstract

**Background:**

In myelodysplastic neoplasms (MDS), the 20q deletion [del(20q)] is a recurrent chromosomal abnormality that it has a high co‐occurrence with *U2AF1* mutations. Nevertheless, the prognostic impact of *U2AF1* in these MDS patients is uncertain and the possible clinical and/or prognostic differences between the mutation type and the mutational burden are also unknown.

**Methods:**

Our study analyzes different molecular variables in 100 MDS patients with isolated del(20q).

**Results & Conclusions:**

We describe the high incidence and negative prognostic impact of *U2AF1* mutations and other alterations such as in *ASXL1* gene to identify prognostic markers that would benefit patients to receive earlier treatment.

## Graphical abstract

U2AF1 mutations and ASXL1 alterations are present in approximately half of myelodysplastic neoplasms (MDS) patients with isolated 20q deletion [del(20q)]. U2AF1 mutations have a negative prognostic impact in MDS with isolated del(20q). U2AF1 mutation burden was found to influence prognosis.

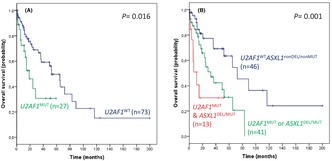

The 20q deletion [del(20q)] is a recurrent chromosomal aberration in myelodysplastic neoplasms (MDS), and as a single abnormality is associated with a favorable outcome according to the Revised International Prognostic Scoring System (IPSS‐R).^1^ *U2AF1* mutations have been associated with chromosomal instability by cell replication obstruction and are frequent (15%–20%) in MDS with del(20q).^2^, ^3^ Nevertheless, their prognostic impact in these MDS patients is uncertain and the possible clinical and/or prognostic differences between mutation type and mutational burden are also unknown.^4^ Other alterations in genes such as *ASXL1*, *SF3B1*, or *SRSF2* may also play a prognostic role in MDS with del(20q).^3^, ^5^

The aim of the current study was to assess the incidence and prognostic impact of *U2AF1* mutations and other gene alterations in MDS patients with isolated del(20q).

From 2001 to 2020, 100 adult MDS patients with isolated del(20q) were studied. The median follow‐up of the series was 17 months (range, 1–199 months). All patient samples were acquired at diagnosis after written informed consent, in compliance with the Declaration of Helsinki and with the approval of the local ethics review committee. As in a previous analysis, *ASXL1, SF3B1, SRSF2*, *U2AF1*, *DNMT3A*, *IDH1*, *IDH2, TP53*, *RUNX1*, and *SETBP1* mutations were analyzed by high‐resolution melting (HRM) and *ASXL1* chromosomal deletion was studied by FISH (Supplemental Methods).^5^ Three quantitative allele‐specific PCR (ASO‐qPCR) were designed to determine variant allele frequency (VAF) and to increase sensitivity in detection of *U2AF1* mutations (S34F, VAF≥0.70%; Q157P, VAF ≥0.15%; Q157R, VAF ≥1.30%; Supplemental Methods). Statistical analysis was generated using the SPSS® statistical data package v.20 and *p* < 0.05 was considered as statistically significant. Pairwise comparisons between patients' characteristics were performed using the Mann–Whitney test for continuous variables and the *χ* ^2^ test or Fisher exact test for categorical variables. Overall survival (OS) was measured from time of diagnosis to last follow‐up or death and was also censored at the time of hematopoietic stem cell transplantation. Survival curves were generated using the Kaplan–Meier method and compared by log‐rank test. A Cox proportional hazard model was constructed for multivariable analysis. Transfusion dependence was defined as patients requiring two or more red blood cells (RBC) transfusions per month or at least one transfusion every 8 weeks in a 4‐month period. Response to azacitidine treatment was assessed using the modified international working group criteria.^6^

Patient characteristics are in Table S2. The median age in the overall series was 74 years and 74% were male patients. According to the IPSS‐R, 74% of patients were categorized as lower‐risk, 15% as intermediate‐risk, and 11% as higher‐risk. Twenty‐four patients without response to first‐line treatment or with higher‐risk MDS received azacitidine. Fourteen patients progressed to acute myeloid leukemia (AML). The median OS for patients was 49 months.

Our approach identified up to 54 patients with a somatic mutation in at least one gene (Figure 1; Table S3). *U2AF1* mutations (*n* = 27) were more frequent in hotspot S34 than Q157 (S34F, *n* = 21; Q157P, *n* = 5; Q157R, *n* = 1) and ASO‐qPCRs determined a mean VAF of 32.8% (range, 0.3% to >44%). *U2AF1* mutations were more frequent in males (89% vs. 68%, *p* = 0.031) and were associated with RBC transfusion dependence (56% vs. 34%, *p* = 0.045; Table S2) and lower OS (median, 19 vs. 52 months, *p* = 0.016; Figure 2A). Patients with *U2AF1* ^Q157^ mutation had a higher percentage of bone marrow blasts and lower OS than *U2AF1* ^S34^ and *U2AF1* ^WT^ patients (median blasts, 6% vs. 2% vs. 1%, *p* = 0.012; median OS, 6 vs. 19 vs. 52 months, *p* = 0.033; Figure S2A). A higher VAF in *U2AF1* mutations was associated with a greater median number of mutations and lower OS compared to a lower VAF or *U2AF1* unmutated (median mutations, 2 vs. 1 vs. 0, *p* < 0.001; median OS, 18 vs. 24 vs. 52 months, for *U2AF1* ^MUT^ VAF >35%, *U2AF1* ^MUT^ VAF <35%, and *U2AF1* ^WT^, respectively, *p* = 0.032).

**FIGURE 1:**
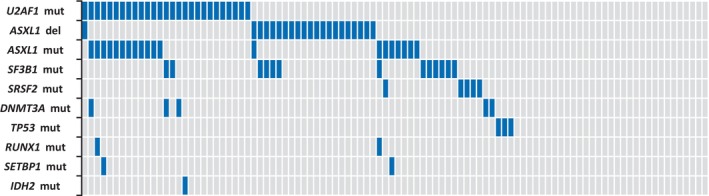
Gene mutations and *ASXL1* chromosomal deletion in the series. Each column represents an individual sample.

**FIGURE 2:**
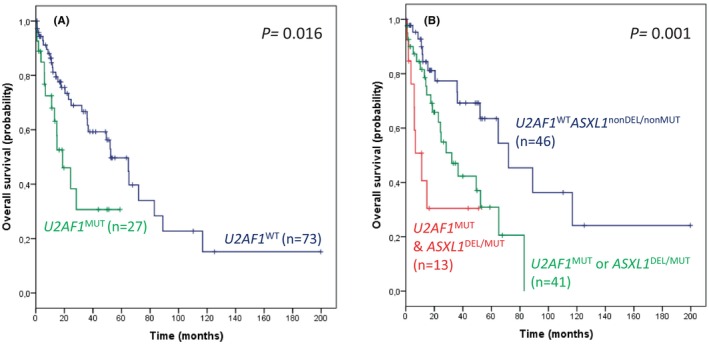
Kaplan–Meier curves. (A) Overall survival (OS) in the series according to *U2AF1* mutations. (B) OS in the series according to the co‐occurrence of *U2AF1* and *ASXL1* alterations.

Regarding other genes, *SF3B1* mutations were associated with a lower hemoglobin level (median, 8.4 vs. 10.4 g/dL, *p* = 0.033), treatment with erythropoiesis‐stimulating agents (ESAs) (85% vs. 29%, *p* < 0.001) and fewer patients in higher‐risk categories of the IPSS‐R (IPSS‐R^HR^) (15% vs. 28%, *p* = 0.012). Patients with *ASXL1* mutations (*n* = 20) showed a lower hemoglobin level (median, 8.2 vs. 10.6 g/dL, *p* = 0.043), and patients with *ASXL1* chromosome deletion (*n* = 21; Figure S3) had a higher progression to AML (29% vs. 10%, *p* = 0.041). Interestingly, patients with *ASXL1* altered either by chromosomal deletion or somatic mutation had lower OS than the remaining patients (median, 24 vs. 65 months, *p* = 0.004; Figure S2B).

Co‐occurrence of *U2AF1* and *ASXL1* alterations was also analyzed, and patients with *U2AF1* mutated and *ASXL1* mutated or deleted (*n* = 13) showed more adverse clinical characteristics and lower OS compared to patients with one (*n* = 41) or neither (*n* = 46) of these two genes altered (IPSS‐R^HR^, 54% vs. 29% vs. 13%, *p* = 0.008; median OS, 11 vs. 32 vs. 72 months, *p* = 0.001; Figure 2B).

The increase in the number of gene mutations was also associated with poor clinical features such as higher ferritin levels and lower OS (median ferritin, 967 vs. 434 vs. 221 ng/mL, *p* = 0.025; median OS, 13 vs. 36 vs. 65 months, for *≥*2, 1, and 0 mutations, respectively, *p* = 0.015; Figure S2C). Additionally, response to azacitidine was analyzed according to clinical and molecular variables, and a lower age remained as the only significant predictor of better response (responders, 53% vs. 11%, for <70 and ≥70 years, respectively, *p* = 0.048).

Gene alterations with a frequency ≥5% were evaluated along with age (≥70 vs. <70 years), sex (female vs. male), IPSS‐R risk groups, and RBC transfusion dependence as candidates in Cox regression modeling. Other variables with no association with OS in the univariable analysis such as treatment with ESAs or azacitidine, were excluded from this analysis. In addition to IPSS‐R and higher age, *U2AF1* mutations and *ASXL1* alterations were independent unfavorable prognostic factors for OS (*U2AF1*, hazard ratio, HR = 2.28; 95% confidence interval, CI: 1.05–4.93; *p* = 0.037; *ASXL1*, HR = 2.86; 95% CI: 1.44–5.67; *p* = 0.003; Table S4).

As in previous analyses, we found a high incidence of *U2AF1* mutations in MDS patients with 20q deletion.^3^, ^5^ In a new finding, *U2AF1* mutations had a negative prognostic impact on the isolated 20q condition. The significance of *U2AF1* mutations in MDS cohorts without del(20q) is variable, in a few cohorts they have no prognostic impact or are confined to low‐risk patients,^7^, ^8^ while in others *U2AF1* mutations show a significant correlation with a worse OS.^4^, ^9^, ^10^ Currently, *U2AF1* mutations are considered detrimental prognostic factors in MDS and have been associated with adverse risk in the new Molecular International Prognostic Scoring System for MDS (IPSS‐M).^10^, ^11^ Furthermore, in our analysis, *U2AF1* ^Q157^ patients had worse clinical parameters and lower OS than *U2AF1* ^S34^ patients, suggesting functional differences between the two mutations based on their different effects in the pre‐mRNA splicing.^4^, ^12^ As in a previous study, *U2AF1* mutation burden was also found to influence prognosis; however, further studies are needed to explore the relationship between high VAF and disease complexity due to an increase in other mutations.^13^

Other splicing genes were also found to be mutated in a significant number of patients, and *SF3B1* mutations in particular were related to anemia, resulting in high dependence of ESAs. On the other hand, *ASXL1* alterations were also frequently detected in MDS with del(20q) and determined a poor clinical outcome.^3^, ^5^ Finally, in concurrence with previous reports, the number of driver mutations was found to provide prognostic information.^14^

The absence of a more comprehensive gene analysis could limit the scope of this study. So, further studies in other MDS with del(20q) cohorts with a more complete gene characterization could be necessary to confirm these novel results.

In summary, risk stratification remains an essential step before treatment decision‐making, and although isolated del(20q) is associated with favorable prognosis in MDS, accompanying gene alterations may have a marked negative impact on clinical outcome. In this context, *U2AF1* mutations and *ASXL1* alterations are present in approximately half of MDS patients with isolated del(20q) and detection at diagnosis would help to identify different subgroups with worse prognosis that could benefit from earlier treatment.

## AUTHOR CONTRIBUTIONS

**Martín I. Castillo:** Conceptualization (equal); data curation (equal); formal analysis (equal); investigation (equal); methodology (equal); writing – original draft (equal). **Villamón E. Ribate:** Investigation (equal); methodology (equal); visualization (equal). **Calabuig M. Muñoz:** Data curation (equal); methodology (equal); supervision (equal). **Sanz G. Santillana:** Conceptualization (equal); supervision (equal). **Such E. Taboada:** Data curation (equal); formal analysis (equal). **Mora E. Casterá:** Data curation (equal); supervision (equal). **Calasanz M. J. Abinzano:** Data curation (equal); supervision (equal). **Irigoyen A. Barranco:** Data curation (equal); methodology (equal). **Collado R. Nieto:** Data curation (equal); methodology (equal); supervision (equal). **Vara M. Pampliega:** Data curation (equal); supervision (equal). **M. L. Blanco:** Data curation (equal); supervision (equal). **Álvarez S. de Andrés:** Data curation (equal); investigation (equal); supervision (equal). **Pérez J. de Oteyza:** Data curation (equal); supervision (equal). **Bernal T. del Castillo:** Data curation (equal); investigation (equal); supervision (equal). **Granada I. Font:** Data curation (equal); formal analysis (equal); methodology (equal). **Jerez A. Cayuela:** Data curation (equal); investigation (equal); methodology (equal); supervision (equal). **M. Díez Campelo:** Conceptualization (equal); investigation (equal); supervision (equal); writing – review and editing (equal). **Abellán R. Sánchez:** Investigation (equal); methodology (equal); visualization (equal). **Solano C. Vercet:** Conceptualization (equal); funding acquisition (equal); investigation (equal); supervision (equal). **Tormo M. Diaz:** Conceptualization (equal); formal analysis (equal); funding acquisition (equal); investigation (equal); supervision (equal); writing – review and editing (equal).

## FUNDING INFORMATION

This work was supported by 2019 3rd Grant of the Spanish Group of Myelodysplastic Syndromes (GESMD).

## CONFLICT OF INTEREST STATEMENT

The authors declare no competing financial interests.

## Supporting information


Data S1.

## Data Availability

N/A.
