# Supplementary material for: Incidence and prognostic impact of U2AF1 mutations and other gene alterations in myelodysplastic neoplasms with isolated 20q deletion
Source: Cancer Med. 2023 Jul 5;12(16):16788–92. doi: 10.1002/cam4.6300 (PMC10501246; doi:10.1002/cam4.6300)
Supplement: Supplementary file 1 — Data S1. [file CAM4-12-16788-s001.docx]

**Incidence and prognostic impact of *U2AF1* mutations and other gene alterations in myelodysplastic neoplasms with isolated 20q deletion.**

**Supplemental methods, tables and figures.**

**1. Supplemental methods.**

**1.1 Design of real-time allele-specific oligonucleotide PCRs (ASO-qPCRs).**

ASO-qPCRs were performed for the three most frequent *U2AF1* mutations (S34F, Q157P, and Q157R), using two reverse primers for each mutation in which the last nucleotide differed at the 3’ position so that they were specific either to the wild-type or the mutated allele. To increase specificity by preventing amplification of the non-matching primers, additional nucleotide mismatches were introduced next to the mutated bases. The primers and TaqMan probes designed along with the annealing temperatures used are shown in **Table S1**. Assays were performed on a LightCycler® 480 (Roche Diagnostics, Indianapolis, USA) and two different PCR reactions were required for each ASO-qPCR: one to detect the mutation (using mutated reverse primer) and the other as a DNA-quality control (using wild-type reverse primer). Each reaction was carried out in a final volume of 10 μl, containing 300 nM of each primer (forward with reverse wild-type or mutated), 200 nM of the probe, 1x of the LightCycler® 480 Probes Master (Roche Diagnostics) and 20 ng of genomic DNA. The amplification program consisted of an initial denaturation step of 10 minutes at 95°C, followed by 50 cycles of 95°C for 15 seconds, 60/67°C for 60 seconds and 72°C for 1 second. Data were analyzed with Gene Scanning Software v1.5 (Roche Diagnostics).

**Table S1.** Primers and TaqMan probes used in each ASO-qPCR.

| **Mutation** | **Sequence (5’-3’)** | **T. annealing (ºC)** |
| --- | --- | --- |
| ***U2AF1*_S34F** | F: TGATAGTGTATGTCATGCTGCTG  Rwt: CGGTTTATTGTGCAACCGAG | 60 |
|  | Rmut: CGGTTTATTGTGCAACC*C*A***A***  Probe: 6FAM-CCATGTGTTTGATATCTTCCCAGC-TAMRA |  |
| ***U2AF1*_Q157P** | F: GTGAAGAAGATGCGGAAAAG  Rwt: CTCACTCACCCCATCTCATACT | 67 |
|  | Rmut: CACTCACCCCATCTCAT*T*C***G***  Probe: 6FAM-TTTAATGGACAGCCGATCCACG-TAMRA |  |
| ***U2AF1*_Q157R** | F: GTGAAGAAGATGCGGAAAAG  Rwt: CTCACTCACCCCATCTCATAC | 60 |
|  | Rmut: CACTCACCCCATCTCAT*T****G***C  Probe: 6FAM-TTTAATGGACAGCCGATCCACG-TAMRA |  |

F: Forward; Rwt: Reverse wild-type; Rmut: Reverse mutated; T: Temperature

**1.2 Sensitivity assays for detection of *U2AF1* mutations.**

Before analyzing patient samples, we performed sensitivity assays to establish the detection limit of the three ASO-qPCRs. We used DNA from three patients with *U2AF1* heterozygous mutations (previously characterized by next generation sequencing and with known variant allele frequencies: S34F, VAF=44%; Q157P, VAF=41%; Q157R, VAF=43%) serially diluted in wild-type DNA to different VAF concentrations: 44%, 22%, 11%, 5.50%, 2.75%, etc. All dilutions were tested in triplicate, including three controls lacking the mutation. This experiment allowed us to generate three standard curves for future VAF quantifications (**Figure S1)**. The technique detection limit was established at 0.70% dilution for S34F mutation, 0.15% dilution for Q157P mutation, and 1.30% dilution for Q157R mutation.


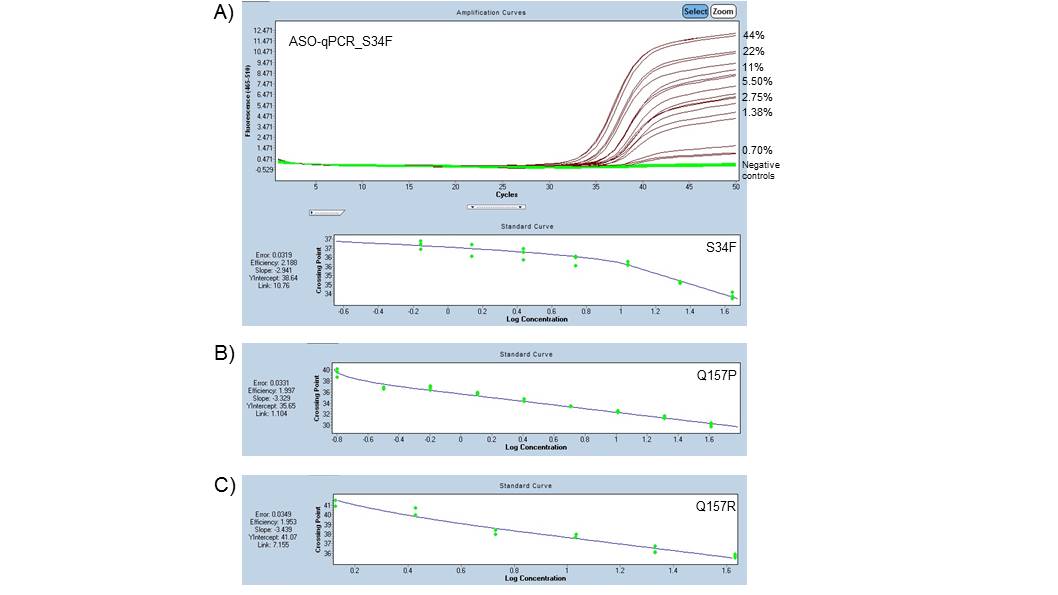


**Figure S1.** ASO-qPCRs assays for detection of *U2AF1* mutations. Plot A shows the PCR amplifications of the serial dilutions for the S34F mutation with the standard curve obtained. Plot B shows the standard curve obtained for the Q157P mutation. Plot C shows the standard curve obtained for the Q157R mutation.

**2. Probes used in FISH study.**

**A)** *ASXL1* probe: aqua signal (20q11; *ASXL1* gene):

***
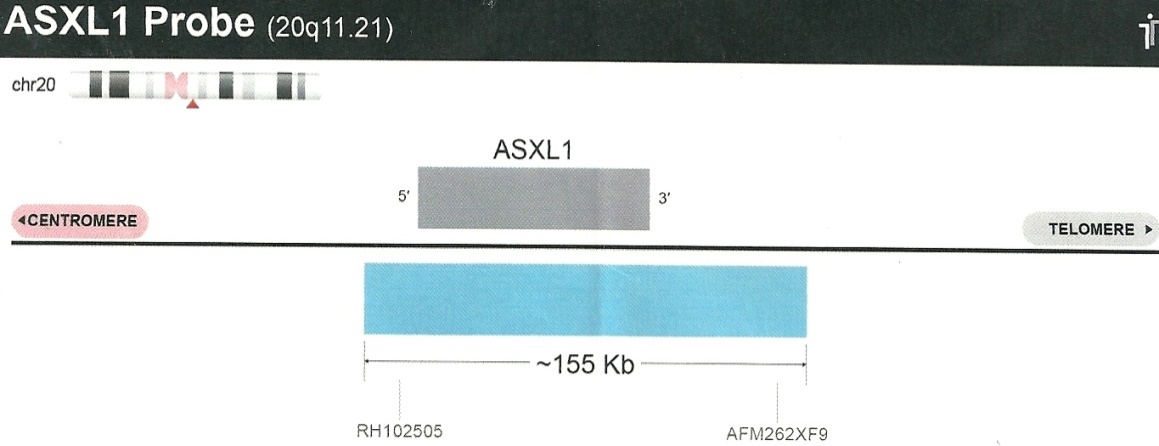
***

**B)** del(20q) probe: orange signal (20q12; *PTPRT* gene) and green signal (20q13; *MYBL2* gene):


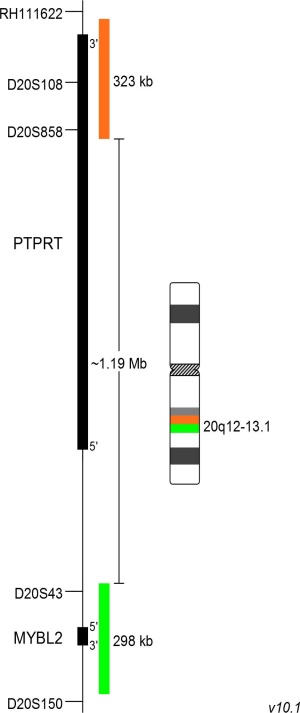


**Table S2.** Characteristics of the global series and according to *U2AF1* mutations with the statistical comparation between mutated and unmutated.

*Range; WHO: World Health Organization; MDS: myelodysplastic neoplasm; LB: low blasts; IB: increased blasts; BM: bone marrow; LDH: lactate dehydrogenase; IPSS-R: Revised International Prognostic Scoring System; FISH: fluorescent in situ hybridization; RBC: red blood cells; IWG: International Working Group criteria; AML: acute myeloid leukemia; allo-HSCT: allogeneic hematopoietic stem cell transplantation; MUT: mutated; WT: wild-type.

**Table S3.** Gene mutations under study.

| **SAMPLE** | **GENE** | **PROTEIN (VAF)** | **CODING** | **VARSOME** | **COSMIC** | **CATEGORIZATION** |
| --- | --- | --- | --- | --- | --- | --- |
| **S1** | *DNMT3A* | p.N797S | c.2390A>G | Likely pathogenic | 7339083 | Oncogenic |
|  | *SF3B1* | p.K700E | c.2098A>G | Pathogenic | 84677 | Oncogenic |
|  | *U2AF1* | p.S34F (>44%) | c.101C>T | Pathogenic | 166866 | Oncogenic |
| **S3** | *SRSF2* | p.P95L | c.284C>T | Pathogenic | 146288 | Oncogenic |
| **S4** | *ASXL1* | p.E635fs | c.1900_1922del | Likely pathogenic | 36165 | Oncogenic |
|  | *U2AF1* | p.Q157P (>41%) | c.470A>C | Pathogenic | 211534 | Oncogenic |
| **S14** | *U2AF1* | p.S34F (42%) | c.101C>T | Pathogenic | 166866 | Oncogenic |
| **S15** | *SRSF2* | p.P95H | c.284C>A | Pathogenic | 211504 | Oncogenic |
| **S16** | *DNMT3A* | p.Q842X | c.2524C>T | Pathogenic | 5752684 | Oncogenic |
| **S21** | *TP53* | p.C238Y | c.713G>A | Likely pathogenic | 11059 | Oncogenic |
| **S22** | *SF3B1* | p.K666N | c.1998G>C | Likely pathogenic | 131557 | Oncogenic |
| **S24** | *SF3B1* | p.K700E | c.2098A>G | Pathogenic | 84677 | Oncogenic |
| **S25** | *ASXL1* | p.G646fs* | c.1926_1927ins | Pathogenic | 914346 | Oncogenic |
|  | *U2AF1* | p.S34F (>44%) | c.101C>T | Pathogenic | 166866 | Oncogenic |
|  | *SETBP1* | p.D868N | c.2602G>A | Likely pathogenic | 1318400 | Oncogenic |
| **S28** | *ASXL1* | S1429Lfs* | c.4286del | Pathogenic | - | Oncogenic |
|  | *DNMT3A* | p.R882H | c.2645G>A | Likely pathogenic | 52944 | Oncogenic |
|  | *U2AF1* | p.S34F (14.8%) | c.101C>T | Pathogenic | 166866 | Oncogenic |
| **S29** | *ASXL1* | p.S1475R | c.4425C>G | Uncertain significance | - | Uncertain |
| **S31** | *SF3B1* | p.K700E | c.2098A>G | Pathogenic | 84677 | Oncogenic |
| **S32** | *U2AF1* | p.S34F (36.2%) | c.101C>T | Likely pathogenic | 166866 | Oncogenic |
|  | *SF3B1* | p.K700E | c.2098A>G | Pathogenic | 84677 | Oncogenic |
| **S33** | *TP53* | p.R248W | c.742C>T | Likely pathogenic | 10656 | Oncogenic |
| **S34** | *U2AF1* | p.S34F (4.3%) | c.101C>T | Pathogenic | 166866 | Oncogenic |
| **S38** | *SF3B1* | p.K700E | c.2098A>G | Pathogenic | 84677 | Oncogenic |
| **S39** | *SF3B1* | p.K700E | c.2098A>G | Pathogenic | 84677 | Oncogenic |
| **S41** | *ASXL1* | p.R693X | c.2077C>T | Pathogenic | 51388 | Oncogenic |
|  | *U2AF1* | p.S34F (41.5%) | c.101C>T | Likely pathogenic | 166866 | Oncogenic |
| **S42** | *SRSF2* | p.P95L | c.284C>T | Pathogenic | 146288 | Oncogenic |
| **S43** | *ASXL1* | p.G646fs* | c.1926_1927ins | Pathogenic | 914346 | Oncogenic |
| **S44** | *ASXL1* | p.G646fs* | c.1926_1927ins | Pathogenic | 914346 | Oncogenic |
|  | *U2AF1* | p.Q157R (19.8%) | c.470A>G | Pathogenic | 211532 | Oncogenic |
|  | *RUNX1* | p.A142Rfs* | c.424_425del | Likely pathogenic | - | Oncogenic |
| **S46** | *U2AF1* | p.Q157P (>41%) | c.470A>C | Pathogenic | 211534 | Oncogenic |
| **S50** | *ASXL1* | p.G646fs* | c.1926_1927ins | Pathogenic | 914346 | Oncogenic |
|  | *SF3B1* | p.K700E | c.2098A>G | Pathogenic | 84677 | Oncogenic |
|  | *RUNX1* | p.R346Pfs* | c.1036_1037ins | Pathogenic | 36063 | Oncogenic |
| **S51** | *ASXL1* | p.G646fs* | c.1926_1927ins | Pathogenic | 914346 | Oncogenic |
|  | *U2AF1* | p.Q157P (>41%) | c.470A>C | Pathogenic | 211534 | Oncogenic |
| **S54** | *U2AF1* | p.S34F (<0.7%) | c.101C>T | Pathogenic | 166866 | Oncogenic |
| **S55** | *U2AF1* | p.S34F (>44%) | c.101C>T | Pathogenic | 166866 | Oncogenic |
| **S56** | *ASXL1* | p.G646fs* | c.1926_1927ins | Pathogenic | 914346 | Oncogenic |
| **S57** | *ASXL1* | p.G646fs* | c.1926_1927ins | Pathogenic | 914346 | Oncogenic |
|  | *U2AF1* | p.S34F (>44%) | c.101C>T | Pathogenic | 166866 | Oncogenic |
| **S59** | *ASXL1* | p.Q910X | c.2728C>T | Likely pathogenic | - | Likely Oncogenic |
|  | *U2AF1* | p.S34F (34.9%) | c.101C>T | Pathogenic | 166866 | Oncogenic |
| **S62** | *ASXL1* | p.T836Ifs* | c.2507del | Likely pathogenic | 97039 | Oncogenic |
| **S64** | *ASXL1* | p.E635fs | c.1900_1922del | Likely pathogenic | 36165 | Oncogenic |
|  | *SRSF2* | p.P95H | c.284C>A | Pathogenic | 211504 | Oncogenic |
| **S65** | *RUNX1* | p.T148Rfs* | c.442_443ins | Pathogenic | 1717871 | Oncogenic |
| **S66** | *DNMT3A* | p.R882H | c.2645G>A | Likely pathogenic | 52944 | Oncogenic |
|  | *U2AF1* | p.S34F (31.4%) | c.101C>T | Pathogenic | 166866 | Oncogenic |
| **S71** | *SF3B1* | p.K700E | c.2098A>G | Pathogenic | 84677 | Oncogenic |
| **S73** | *IDH2* | p.R140Q | c.419G>A | Pathogenic | 41590 | Oncogenic |
|  | *U2AF1* | p.S34F (>44%) | c.101C>T | Pathogenic | 166866 | Oncogenic |

| **SAMPLE** | **GENE** | **PROTEIN (VAF)** | **CODING** | **VARSOME** | **COSMIC** | **CATEGORIZATION** |
| --- | --- | --- | --- | --- | --- | --- |
| **S76** | *SF3B1* | p.K666Q | c.1996A>C | Pathogenic | 132950 | Oncogenic |
| **S77** | *ASXL1* | G710Efs* | c.2127del | Likely pathogenic | 5990080 | Oncogenic |
|  | *U2AF1* | p.S34F (>44%) | c.101C>T | Pathogenic | 166866 | Oncogenic |
| **S79** | *U2AF1* | p.S34F (3.8%) | c.101C>T | Pathogenic | 166866 | Oncogenic |
| **S80** | *SF3B1* | p.K700E | c.2098A>G | Pathogenic | 84677 | Oncogenic |
| **S81** | *DNMT3A* | p.R882H | c.2645G>A | Likely pathogenic | 52944 | Oncogenic |
| **S82** | *TP53* | p.G187V | c.560G>T | Likely pathogenic | 45240 | Oncogenic |
| **S84** | *U2AF1* | p.Q157P (0.28%) | c.470A>C | Pathogenic | 211534 | Oncogenic |
| **S85** | *SF3B1* | p.K700E | c.2098A>G | Pathogenic | 84677 | Oncogenic |
| **S87** | *U2AF1* | p.S34F (41.6%) | c.101C>T | Pathogenic | 166866 | Oncogenic |
| **S88** | *ASXL1* | p.G646fs* | c.1926_1927ins | Pathogenic | 914346 | Oncogenic |
|  | *U2AF1* | p.S34F (40.6%) | c.101C>T | Pathogenic | 166866 | Oncogenic |
| **S90** | *ASXL1* | p.G646fs* | c.1926_1927ins | Pathogenic | 914346 | Oncogenic |
| **S92** | *ASXL1* | p.Q778X | c.2332C>T | Pathogenic | 133576 | Oncogenic |
|  | *U2AF1* | p.S34F (>44%) | c.101C>T | Pathogenic | 166866 | Oncogenic |
| **S93** | *U2AF1* | p.S34F (>44%) | c.101C>T | Pathogenic | 166866 | Oncogenic |
| **S94** | *U2AF1* | p.S34F (24.6%) | c.101C>T | Pathogenic | 166866 | Oncogenic |
| **S97** | *ASXL1* | p.L817X | c.2448del | Pathogenic | 1716556 | Oncogenic |
|  | *SETBP1* | p.S869G | c.2605A>G | Likely pathogenic | 1666674 | Oncogenic |
| **S98** | *SF3B1* | p.K700E | c.2098A>G | Pathogenic | 84677 | Oncogenic |
| **S99** | *ASXL1* | R634* | c.1900A>T | Uncertain significance | 1012900 | Likely Oncogenic |
|  | *U2AF1* | p.Q157P (35.3%) | c.470A>C | Pathogenic | 211534 | Oncogenic |
| **S100** | *U2AF1* | p.S34F (40.0%) | c.101C>T | Pathogenic | 166866 | Oncogenic |

**
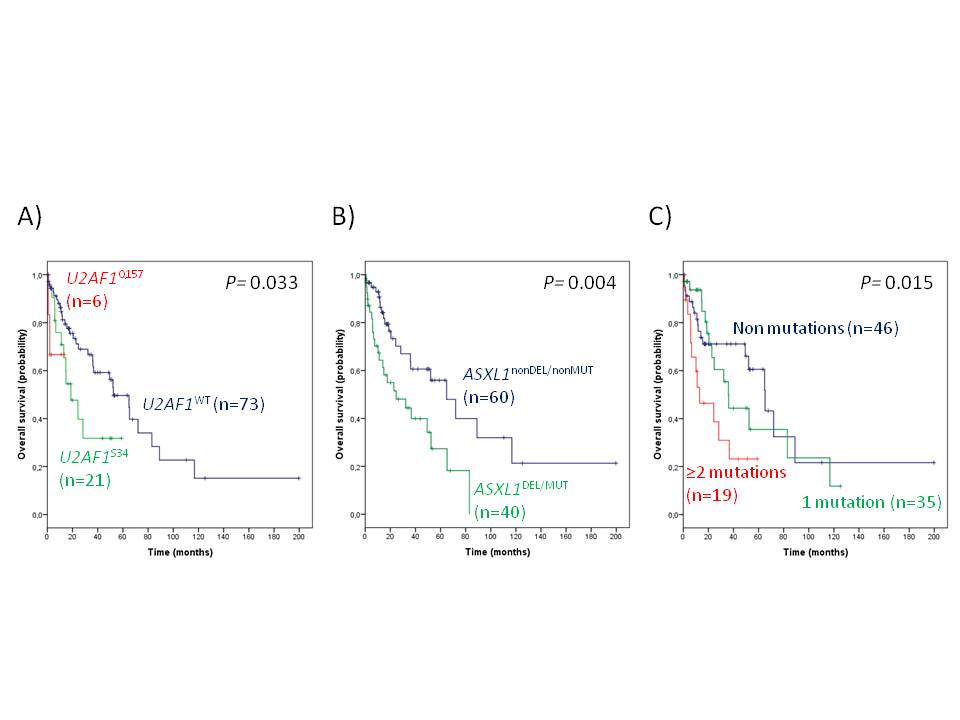
**

**Figure S2.** Kaplan–Meier curves. **(A)** Overall survival (OS) in the series according to the type of *U2AF1* mutation. **(B)** OS in the series according to *ASXL1* alterations. **(C)** OS in the series according to the number of gene mutations.


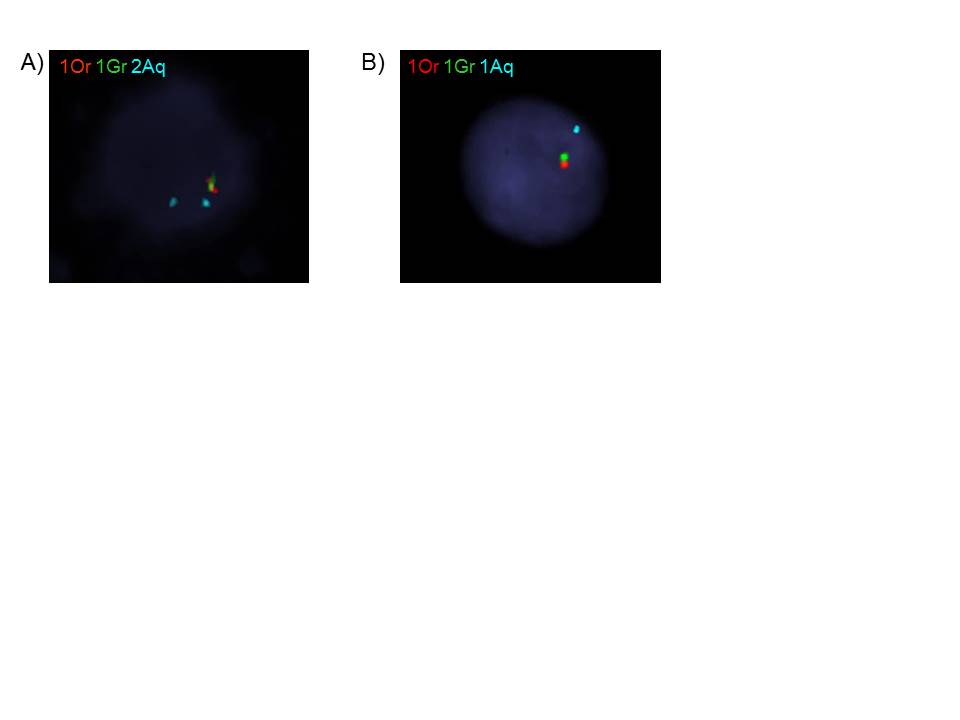


**Figure S3.** FISH analysis of *ASXL1* chromosomal region: orange signal, *PTPRT* gene; green signal, *MYBL2* gene; aqua signal, *ASXL1* gene. (A) del(20q) with 2 copies of *ASXL1* gene. (B) del(20q) with *ASXL1* heterozygous deletion.

**Table S4.** Multivariable analysis by Cox regression for overall survival.

| **Variable** | **Overall survival** | | |
| --- | --- | --- | --- |
|  | **HR** | **CI 95%** | ***P-value*** |
| **IPSS-R:** |  |  |  |
| **Intermediate *vs.* Low/very low** | **2.88** | 1.75 – 4.74 | **<0.001** |
| **High/Very high *vs.* Low/very low** | **4.78** | 1.59 – 14.37 | **0.005** |
| **Age, ≥70 *vs.* <70 years** | **2.19** | 1.07 – 4.48 | **0.031** |
| **Sex, female *vs.* male** | 0.58 | 0.25 – 1.38 | 0.22 |
| **RBC transfusion dependence** | 1.07 | 0.56 – 2.05 | 0.84 |
| ***U2AF1*^MUT^ *vs.* *U2AF1*^nonMUT^** | **2.28** | 1.05 – 4.93 | **0.037** |
| ***SF3B1*^MUT^ *vs.* *SF3B1*^nonMUT^** | 0.55 | 0.23 – 1.32 | 0.18 |
| ***SRSF2*^MUT^ *vs.* *SRSF2*^nonMUT^** | 1.06 | 0.12 – 9.05 | 0.96 |
| ***DNMT3A*^MUT^ *vs.* *DNMT3A*^nonMUT^** | 0.31 | 0.05 – 1.80 | 0.19 |
| ***ASXL1*^DEL/MUT^ *vs.* *ASXL1*^nonDEL/nonMUT^** | **2.86** | 1.44 – 5.67 | **0.003** |

HR, hazard ratio; CI, confidence interval; IPSS-R: Revised International Prognostic Scoring System; RBC: red blood cells.
